# Fossils with Feathers and Philosophy of Science

**DOI:** 10.1093/sysbio/syz010

**Published:** 2019-02-11

**Authors:** Joyce C Havstad, N Adam Smith

**Affiliations:** 1 Department of Philosophy, Oakland University, 146 Library Drive, Rochester, MI 48309, USA; 2 Campbell Geology Museum, Clemson University, 140 Discovery Lane, Clemson SC 29634, USA

**Keywords:** Aves, avian origins, Kuhn, Lakatos, Popper, theropod hypothesis

## Abstract

The last half century of paleornithological research has transformed the way that biologists perceive the evolutionary history of birds. This transformation has been driven, since 1969, by a series of exciting fossil discoveries combined with intense scientific debate over how best to interpret these discoveries. Ideally, as evidence accrues and results accumulate, interpretive scientific agreement forms. But this has not entirely happened in the debate over avian origins: the accumulation of scientific evidence and analyses has had some effect, but not a conclusive one, in terms of resolving the question of avian origins. Although the majority of biologists have come to accept that birds are dinosaurs, there is lingering and, in some quarters, strident opposition to this view. In order to both understand the ongoing disagreement about avian origins and generate a prediction about the future of the debate, here we use a revised model of scientific practice to assess the current and historical state of play surrounding the topic of bird evolutionary origins. Many scientists are familiar with the metascientific scholars Sir Karl Popper and Thomas Kuhn, and these are the primary figures that have been appealed to so far, in prior attempts to assess the dispute. But we demonstrate that a variation of Imre Lakatos’s model of progressive versus degenerative research programmes provides a novel and productive assessment of the debate. We establish that a refurbished Lakatosian account both explains the intractability of the dispute and predicts a likely outcome for the debate about avian origins. In short, here, we offer a metascientific tool for rationally assessing competing theories—one that allows researchers involved in seemingly intractable scientific disputes to advance their debates.

## Competing Standards of Science

Why is it so difficult to separate the good scientific wheat from the bad scientific chaff? The answer to this question is two-fold. First, there are many standards to meet when practicing good science. Second, meeting them often requires activities which can be cast in either a positive or negative light. For instance: evidence seeking is a typical scientific activity. But seeking evidence whether or not it turns out to support one’s preferred scientific theory is often indistinguishable, in practice, from seeking evidence in support of one’s preferred scientific theory. The former meets the standards of good science but the latter does not. When a scientist has gathered evidence in support of their preferred scientific theory, observers often cannot tell whether the evidence was gathered in the good evidence-seeking way or the bad. In ambiguous cases, proponents of the theory are liable to view the process as corroborative, while opponents are liable to view it as verificationist. Another example is that of theory evolution. Those who endorse a theory will likely see development and modification of the theory as progressive; but critics may interpret these changes as excessively revisionist. Finally, consider the notion of scientific agreement. When proponents of a debate are happy with the establishment of an agreement, they call it a consensus. When unhappy, they label it dogma. For each of these antagonisms, there is some practice that seems to be associated with good science—something that might help to characterize it, such as evidence gathering or theory evolution or scientific agreement—but then it turns out that it is the *good* form of that practice which is associated with good science, not the practice itself. Good scientific practice makes for good science! This dictum is not, by itself, exceedingly diagnostic.

No single or simple standard for assessing good science has yet been proposed and validated by widespread, successful application. Sir Karl Popper’s criterion of falsifiability (Popper 1934/1959) is a favored candidate, but the problems with that standard are well known, and significant enough to have driven many to accept Thomas Kuhn’s alternative position that scientific theory choice is not rationally determined (Kuhn 1962; more on the interplay between Popper and Kuhn in the next section). It would be nice if there were another option for assessing scientific quality—something to supplement the overly simplistic Popperian position and compete with the excessively populist Kuhnian stance. Here, we offer just such an alternative, by reviving and rehabilitating Imre Lakatos’ account of progressive as opposed to degenerative research programmes (Lakatos 1968, 1970).

Our proximate aim in revisiting the Lakatosian method for assessing scientific practice is to advance the debate about avian origins. The currently received view amongst evolutionary biologists is that birds are dinosaurs—specifically, that they are maniraptoran theropods. The ascendance of this view was initiated by John Ostrom’s (1969) discovery of the bird-like theropod *Deinonychus antirrhopus* (Ostrom 1969), and advanced in a trio of publications rapidly following that discovery (Ostrom 1973, 1974, 1976). Still, not everyone is today convinced that birds are descended from maniraptoran theropods. The notion has historically encountered skepticism (Tarsitano and Hecht 1980; Bock 1986; Olson 1987; Martin 1991) and there is at least one strain of ongoing, fervent opposition (Feduccia 1980, 1996, 2002, 2012). Observers have repeatedly cautioned against calling the debate over (Olson 2002; Zhou 2004).

In 2002, the preceding 25 years of debate were wryly described as divisive (Prum 2002). That same year, the fervor surrounding Ostrom’s theropod hypothesis led one commentator to dub it the Birds-Are-Dinosaurs-Movement, or BADM (Olson 2002). Since then, discussion between BADM and its opposition—the notion that Birds-Are-Not-Dinosaurs, or BAND—has turned downright acrimonious (Feduccia 2002, 2013, 2016; Prum 2003; Smith et al. 2015). All this despite the fact that, in the time between Ostrom’s 1969 discovery of *D. antirrhopus* and now, many purportedly key pieces of evidence have been discovered (Barsbold 1983; Qiang et al. 1998; Xu et al. 2003; Lipkin et al. 2007; Nesbitt et al. 2009; Xu et al. 2009; Xing et al. 2016; Smithwick et al. 2017; McNamara et al. 2018), several supposedly comprehensive analyses have been conducted (Chiappe 1995; Turner et al. 2012; Clarke 2013; Brusatte et al. 2014), and various pleading appeals to scientific virtue have been made (Feduccia 2002, 2013, 2016; Prum 2003; Smith et al. 2015). The dispute has lingered, while the discussion has soured.

So, here, we examine an unexpected but major source of ensuing bitterness, we revive and rehabilitate Lakatos’ unfortunately neglected account of (dys)functional scientific practice, and we develop and apply that account to a historical review of the BADM versus BAND debate in a diagnostic manner. We caution against conflating merely static or even degenerative scientific practice with unscientific practice, and conclude with a prediction generated by the Lakatosian model of scientific practice regarding the likely fates of BADM and BAND. Although the idea that birds are dinosaurs is currently at the core of a progressive Lakatosian research programme, the idea that birds are not dinosaurs has become, at best, the core of a static research programme and is, at worst, a degenerative one. Our ultimate aim is to demonstrate how Lakatos’ model can be applied not just here, but elsewhere—wherever contradictory, contested assessments of research quality are impeding progress and increasing hostility in scientific debate.

## Failures of Falsifiability and Irrationality

A significant attitudinal shift in the modern debate about avian origins occurred in 2003, when one disputant (Prum 2003) accused another (Feduccia 2002) of maintaining a position that could no longer be reasonably characterized as scientific, on the grounds that the position was so vague as to be unfalsifiable. According to this critique, scientific theories must meet the minimal standard of falsifiability in order to qualify as scientific. This way of thinking—initiated by the philosopher of science Sir Karl Popper (1934/1959)—tends to be a popular one in science.

Despite how simple and elegant a solution to the problem of demarcating science from nonscience the falsifiability criterion appears to be, there are problems with it. The most significant problem is that only rarely does the practice of science consist in assessing theories by isolating hypotheses and testing them in fortuitous conditions where everything else is fixed. Hypotheses are designed to test scientific theories, and scientific theories are often vague and underdeveloped, especially in their early stages. A scientist with a nascent theory may need to be able to develop speculative hypotheses, test them, and reject the ones that do not pan out without having to reject the entire underlying theory. Or, a scientist might need to call into question another aspect of the experimental framework in response to a failed test, holding onto both that hypothesis and the underlying theory—just in case what they have really discovered is a previously undetected problem with the testing conditions, or an insufficiently examined background assumption. Using falsifiability as a minimal standard for scientific practice fails to accommodate evolution and uncertainty in theory and testing conditions. We need an account of scientific practice that allows for the development of theories and tests via trial and error, but which also allows us to say that at some point, enough is enough—that the pursuit of a once-scientific idea has given way to dogmatism.

Popper recognized the import of revision in science, objecting only to the degenerate form of it that he called *ad hoc* revisionism. The problem is in discriminating between these two—principled and ad hoc revisionism—and Popper himself did not ever manage to craft a compelling solution to this problem. This is where the Hungarian-born Imre Lakatos, another philosopher of science and eventual émigré to Britain, comes into the picture. Scientists tend to be much more familiar with Thomas Kuhn’s (1962) response to the problem of theory choice raised by the failure of Popper’s simple falsificationism, as this is where the notion of paradigm shift comes from. But Kuhn’s so-called solution to the problem makes theory “choice” irrational: he says that what makes a scientist stick with a theory despite its failures and anomalies (staying within the paradigm), or what makes a scientist decide after a series of discrepancies that enough is enough (shifting the paradigm), is not a choice at all. Rather, it is a psychologistic and communal verdict: the result of a feeling or a mood the scientist has, related to a moment in the scientific community of appeal or lack thereof. What Lakatos (1968, 1970) offers instead is a promising alternative solution to the Popperian problem of scientific theory choice: one that can provide a rational rather than a populist account of how to discriminate between principled and ad hoc revisionism.

**Figure 1.**
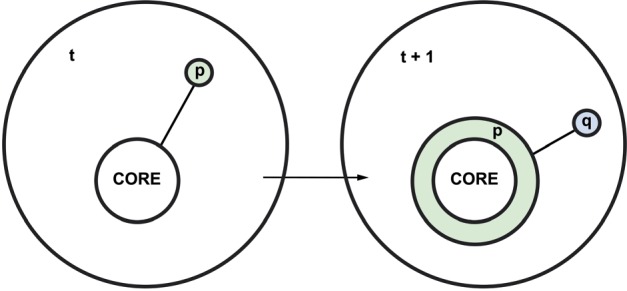
Graphical representation of how Lakatos (1968, 1970) conceived of research programmes. On the left, a research programme at time “t” consists of its core commitment(s) and one empirical, speculative postulate “p” (which extends out from the core in a lightly colored bubble). On the right, at time t + 1, the empirical postulate “p” has been confirmed (adding a protective belt of its color around the core), and a new postulate “q” (in a differently colored bubble) is being considered for testing.

At the foundation of Lakatos’ account of rational theory choice is the idea that scientific practice consists largely of interaction between competing research programmes, rather than merely isolated hypotheses or even theories. See Figure 1 for a depiction of the Lakatosian idea of a research programme.

A scientific research programme is, in its most basic form, a commitment to something. Initially, this might be something as vague as the idea that “birds are descended from dinosaurs” or that “birds are not descended from dinosaurs—they are descended from an independent line of more basal archosaurs instead.” It is not always easy to tell, especially at first, exactly which of those proposals being considered by a research programme are the commitments that belong inside the core, and which are mere postulates that—if they survive testing—can be added to the empirical content of the programme, or—if they don’t survive testing—can simply be discarded.

Regardless, the internal commitments ultimately form what Lakatos called the *hard core* of a research programme. These are those postulates that end up defining the scientific position being explored and defended by that particular programme. The process of exploration and defense suggested by the core forms what Lakatos called the *positive heuristic* of a research program. This is a set of hints and anomalies which suggest how to develop the core in a progressive rather than degenerative way. The positive heuristic of a research programme encourages development of speculative hypotheses about empirical circumstances that are implied by the commitments of the core, but which have not yet been satisfactorily explained, established, or discerned. When such hypotheses can be tested, and if such testing corroborates the corresponding circumstances, then this adds empirical content to the core. Building up corroborated content gradually forms what Lakatos called a *protective belt* around the core of a research programme. Failures to test some of a research programme’s speculative hypotheses, or failures to corroborate the circumstances postulated by such hypotheses, are acceptable failures on the Lakatosian view of scientific practice—as long as alternative hypotheses can eventually be developed and tested, and at least some corroboration is intermittently acquired. Threats to the core itself are the only ones that cannot be tolerated, and this commitment is what Lakatos called the *negative heuristic* of a research programme. When introducing his concept of research programmes, Lakatos offered the example of Cartesian metaphysics as an illustration. This research programme was committed to a clockwork-driven, push-motion-based, mechanistic theory of the universe. Its negative heuristic was to avoid inconsistent theories (such as Newton’s theory of action at a distance) and its positive heuristic was to look for mechanical explanations of phenomena (Lakatos 1968, p. 168).

Unfortunately, Lakatos first articulated his solution to the problem of rational theory choice in late 1968, elaborated the account in 1970, and then unexpectedly died of a heart attack in early 1974, at the age of only 51 years. So, his account, which was born neither complete nor perfect, did not itself benefit from an extended period of exploration, revision, expansion, and refinement by its creator. Our task now is to explore various implications of the account, address some of its readily apparent flaws, and show how a refurbished version of the account can be helpfully applied to—for instance—the ongoing and seemingly intractable avian origins debate.

## Revisiting the Lakatosian Model

The first thing to notice about Lakatos’ account of rational theory choice in scientific practice is that it is not actually an apt tool for distinguishing science from nonscience. In other words, the account does not itself provide a compelling solution to the demarcation problem which has fascinated Popper and so many others. But this aspect of Lakatos’ account is an asset rather than a flaw. What Lakatos’ account is aptly designed to do is separate good science from bad science, and securing that distinction is just as important as solving the demarcation problem. Conflating these two issues—that of distinguishing science from nonscience and good science from bad science—has been remarkably unhelpful. One might say a similar thing about the frequent conflation of questions about avian origins and the evolution of flight. It is not as if all science is good science, and all bad science is nonscience. Once that fact has been granted, it is clear that we require both a way of demarcating science from nonscience and a way of distinguishing good science from bad science. We might also want, within the category of nonscience, a way of demarcating plainly nonscientific practice from pseudoscientific practice (that which insidiously imitates science). We might further want, within the category of bad science, a way of distinguishing merely imprudent practices (e.g., small sample sizes) from pernicious ones (also known as junk science). And within the category of junk science, we might want to discriminate between spurious science (e.g., data dredging) and fake science (e.g., data fabrication). One virtue of having a more nuanced framework—one that distinguishes beyond the binary of good science and nonscience—is that having such a framework makes it possible to critique bad science, while nonetheless admitting that at least some of it is still scientific.

Given the preexisting need for a new and more exacting framework, and while recalling the remarkably antagonistic character of the recent debate between BADM and BAND, neither is it hard to understand how plausible objections to contentious scientific practice might have been mistakenly expressed as accusations of nonscientific practice (Prum 2003); nor is it hard to imagine why such accusations might have unproductively escalated rather than advanced the debate (Feduccia 2013, 2016; Smith et al. 2015). No well-trained scientist engaged in good-faith scientific research at a recognizably scientific institution wants to be accused of doing nonscientific work by other scientists merely because they are engaged in a long-running scientific disagreement (Ostrom 1969; Gauthier 1986; Smith et al. 2015; Feduccia 2016). Even scientists not directly involved in such a debate are likely to resist calls to excise disputants on one side (Olson 2002), and those not yet committed to a side will reasonably object to declarations that the debate is over (Zhou 2004)—especially if it looks as if this move is being made in a nonscientific or even just a metascientific way.

So, let us grant that scientific practice simply is whatever it is that those who are typically called scientists at what are typically called scientific institutions do that is typically called scientific. Philosophers of science have offered other assessments (e.g., Laudan 1983; Longino 1990) relevant to how one might respond to the demarcation problem—precisely understood as how to demarcate science from nonscience, regardless of what distinguishes good science from bad—but the simple sociological postulate will suffice for our purposes. Again, Lakatos’ account of research programmes presupposes a preexisting account of scientific research; his position assumes that we can already identify what counts as science. Here, we are moving past that feature of his account by provisionally positing that scientific practice is, generally, what scientists do at scientific institutions that gets called scientific. Usually, this involves developing, testing, and defending postulates in an especially empirical way, though it can involve purely theoretical exploration in some areas of science. Examples include disciplines such as string theory that may not yet be subject to instrumental investigation, as well as branches of historical science that are primarily engaged in data collection. With this working notion of scientific practice in play, Lakatos’ account can help distinguish progressive research programmes from degenerative ones—a distinction we can use for competing theories of avian origins.

## The Evolution of Birds

For the last 50 or so years, there has been growing agreement that birds are descended from dinosaurs—specifically, from maniraptoran theropods. This consensus (if you are in favor) or dogma (if you are not) was sparked by John Ostrom’s (1969) discovery of the bird-like theropod *D. antirrhopus* (Ostrom 1969). In a series of follow-up publications, Ostrom suggested that: instead of being descended from more basal archosaurs, birds were coelurosaurs (Ostrom 1973); that there was a cursorial or ground-up origin for avian flight (Ostrom 1974); and that, even more specifically, birds were maniraptoran theropods (Ostrom 1976). There were alternative hypotheses for avian origins available at the time: Walker (1972), for instance, had relied on a specimen of *Sphenosuchus* to argue that birds were crocodylomorphs. And not all of Ostrom’s postulates have entirely withstood scientific scrutiny: today more paleontologists favor the idea of an arboreal origin for flight (Feduccia 1980; Bock 1985, 1986; Norberg 1990), for example, than do the cursorial origin hypothesis. But Ostrom’s placement of an origin for birds within maniraptoran theropods quickly found phylogenetic support (Bakker and Galton 1974; Thulborn 1975; Cracraft 1977; Gauthier and Padian 1985; Gauthier 1986), and that support has so far withstood the test of relatively recent scientific time (Chiappe 1995; Padian 2001; Clarke 2013). A spate of fossil discoveries made over the ensuing decades has only extended the nature of the paleontological agreement about avian theropod origins. Especially important finds include: furculae (fused clavicles) in both avian and nonavian theropods (Barsbold 1983; Lipkin et al. 2007; Nesbitt et al. 2009); feathered dinosaurs, both avian and nonavian (Qiang et al. 1998; Xu et al. 2003; Xing et al. 2016); a transitional hand between the avian and dinosaurian (Xu et al. 2009); and preserved avian and dinosaurian feathers and skin (Smithwick et al. 2017; McNamara et al. 2018).

Prior to the (still reigning) ascension of Ostrom’s theropod hypothesis for avian origins, there was an alternative paleontological agreement about avian origins: birds were descended from other, rather more basal archosaurs. The dominance of this view is generally credited to the success of Gerald Heilmann’s *The Origin of Birds* (1926), in which Heilmann argued that the absence of either a clavicle or a furcula in dinosaurs, plus the presence of a furcula in birds, along with Dollo’s law of irreversibility (i.e., if birds and other archosaurs have furculae but dinosaurs did not, then birds could not be dinosaurs), altogether entailed that birds cannot be descended from dinosaurs. This is why the discovery of dinosaurian furculae (mentioned just above) was especially important to the avian origins debate. Because such fossils were unknown at the time, Heilmann identified pseudosuchians rather than coelosaurians as more likely ancestors of birds. Despite the overwhelming resemblance of birds and small theropod dinosaurs noted by Heilmann, the incompleteness of the fossil record alone forced Heilmann to conclude that “it is evident that all our *requirements of a bird-ancestor are met by the Pseudosuchians, and nothing in their structure militates against the view that one of them might have been the ancestor of the birds.* This of course does not prove that this ancestor was one of the known Pseudosuchians” (Heilmann 1926, p. 191). Heilmann considered the specimens of *Ornithosuchus woodwardi* (Newton 1894) and *Euparkeria capensis* (Broom 1913) to be especially promising indicators of what the ancestor of birds might have looked like.

Prior to the (temporary) ascension of Heilmann’s pseudosuchian hypothesis, the state of play is perhaps best described as one of a lack of agreement about avian origins. Just 2 years after the 1859 publication of Charles Darwin’s *On the Origin of Species by Means of Natural Selection*, the German paleontologist Christian Erich Hermann von Meyer reported the discovery of a single feather of *Archaeopteryx lithographica* (officially described in von Meyer 1862). Two skeletal *Archaeopteryx* specimens were found shortly thereafter, again in Jurassic limestone quarried in Bavaria, and each were eventually described as distinct *Archaeopteryx* species—the London specimen as *Archaeopteryx macrura* (Owen 1863) and the Berlin specimen as *Archaeopteryx siemensii* (Dames 1897). By 1867, Thomas Henry Huxley had already incorporated *Archaeopteryx* into his “On the Classification of Birds” (Huxley 1867), and on 7 February 1868, Huxley delivered a lecture to the Royal Institution of Great Britain entitled “On the Animals Which Are Most Nearly Intermediate Between Birds and Reptiles,” eventually published in London’s *Popular Science Review* (Huxley 1868). In his lecture, Huxley suggested a dinosaurian origin for birds—employing *Archaeopteryx* in his argument, but relying even more heavily on another dinosaurian specimen from the Solnhofen slates, that of *Compsognathus longipes*.

On this issue as well as others, Richard Owen disagreed with Huxley. Owen claimed that apparent similarities between birds and dinosaurs were merely the result of convergence (Owen 1875). Although this dispute between Huxley and Owen was well publicized and much discussed, such scrutiny did not produce any sort of conclusive resolution. During his 30 August 1877, address to the *American Association for the Advancement of Science*, for instance, the American paleontologist Othniel Charles Marsh adopted Huxley’s view of a dinosaurian origin for birds (Marsh 1877). But in the *Kansas City Review of Science and Industry*, Marsh’s compatriot Benjamin Franklin Mudge supported Owen’s alternative position (Mudge 1879). Mudge’s contribution was titled “Are Birds Derived from Dinosaurs?” and his answer to that question was “no” (Mudge 1879). Shortly thereafter an entomologist named Samuel Wendell Williston asked the same question in the same publication and made a compelling but succinct argument that the answer was in fact “yes” (Williston 1879). Williston’s prescient contribution did not resolve the debate, however, and it is probably fair to say that, at the time, there was insufficient evidence available to settle the issue of avian origins. (For more on this phase of the avian origins debate, see Witmer 1991).

In sum, the history of the modern evolutionary debate about the origin of birds can be partitioned into three distinct phases: an initial phase (1859–1925), characterized by a lack of paleontological agreement; and intermediate phase (1926–1968), characterized by widespread paleontological agreement that birds were reptiles but not dinosaurs; and the latest phase (1969–current), which is characterized by widespread paleontological agreement that birds are in fact descended from dinosaurs. What we need to ascertain now is whether this history is one of mere variation in opinion, or advancement of science—and the Lakatosian framework can help.

Figure 2 presents a visual representation of the history of the avian origins debate, accompanied by symbolic drawings of key specimens and ideas, as well as the beginnings of a graphical application of the Lakatosian framework to that debate. Surveying the avian origins debate in this thorough and inclusive way allows us to compare and contrast the Lakatosian status of both the BADM and the BAND camps at different moments in the scientific history of the dispute. In the earlier stages of the debate, both positions represented core commitments of progressive research programmes. But in the current circumstances, the two positions are not so evenly matched.

**Figure 2.**
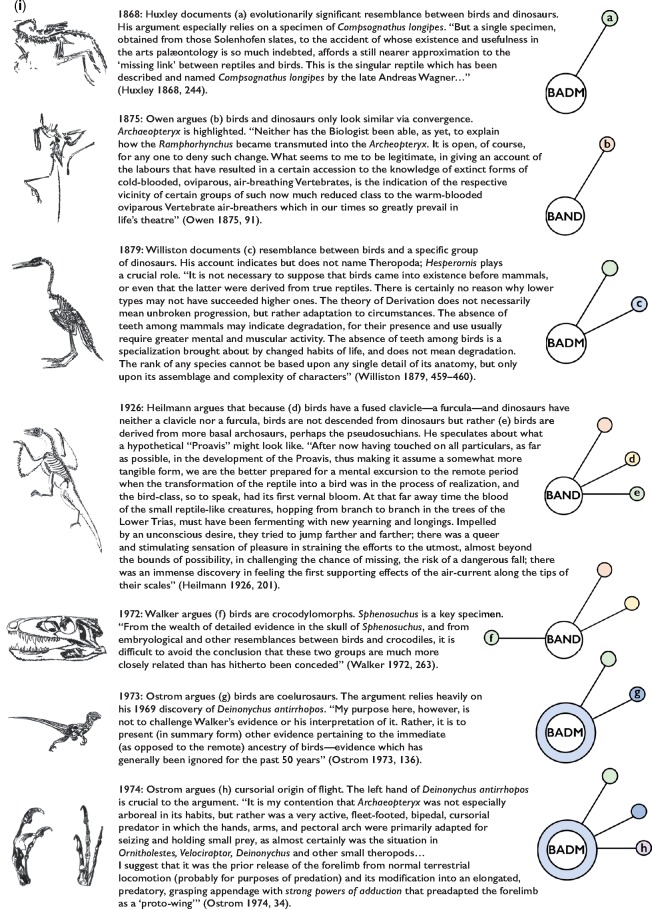

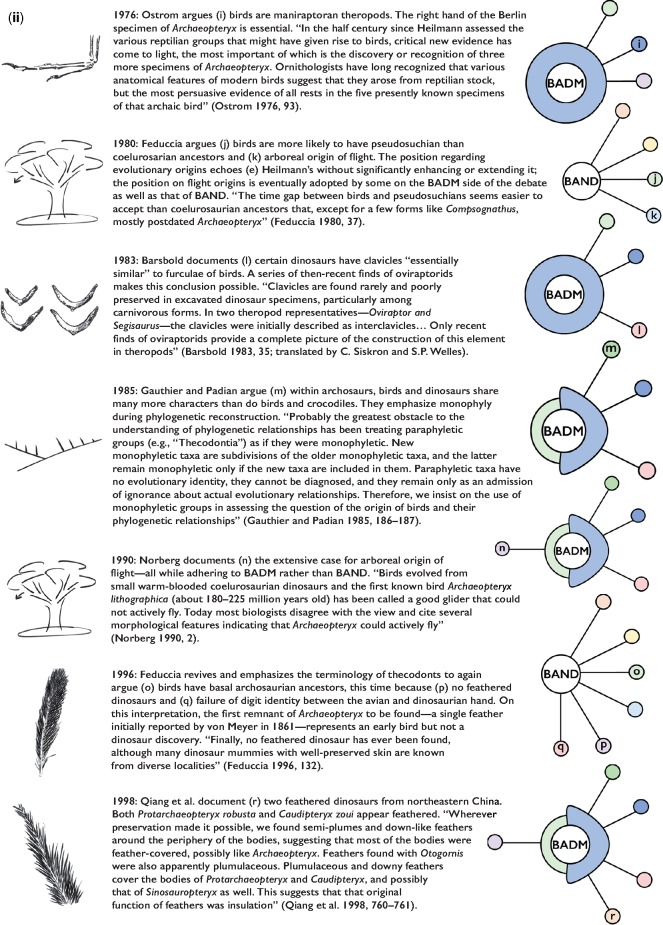
Chronological representation of key moments, ideas, evidence, and theoretical advances from the past 160 years of scientific debate concerning the evolutionary origin of birds. Contributions are marked by year; each pairs a drawing (on the left) indicating a crucial specimen or concept for the contribution with a theoretical visualization (on the right) of how, in a Lakatosian sense, the contribution advances its research program. Commitments to BADM and BAND each form the hard core of their own research programmes. New postulates made by a research program are indicated by the extension of an arm out from the core in a new position around it (and lettered). Opposing postulates, when offered, appear on the opposite position around the core. When a new contribution corroborates a prior postulate, a protective belt of that postulate’s color is added to and surrounds the core (or, in successive stages, the existing belt swells in size and darkens in color). Corroboration is often accompanied by proposal of a more advanced, specific, or developed version of the initial postulate. New postulates that further a line of reasoning in this way appear in the same position as the prior postulate and with the same color scheme, but each advance within such a chain is indicated by a darkening of the associated color. On this way of visually representing Lakatosian research dynamics, colored pieces surrounding the core, differently colored pieces surrounding the core, larger pieces, and darker colored pieces all indicate a healthy program. Cores without protective pieces around them, cores surrounded only by lighter colors, and postulate arms reappearing without darkening, are markers of an ailing program.

**Figure 2.**
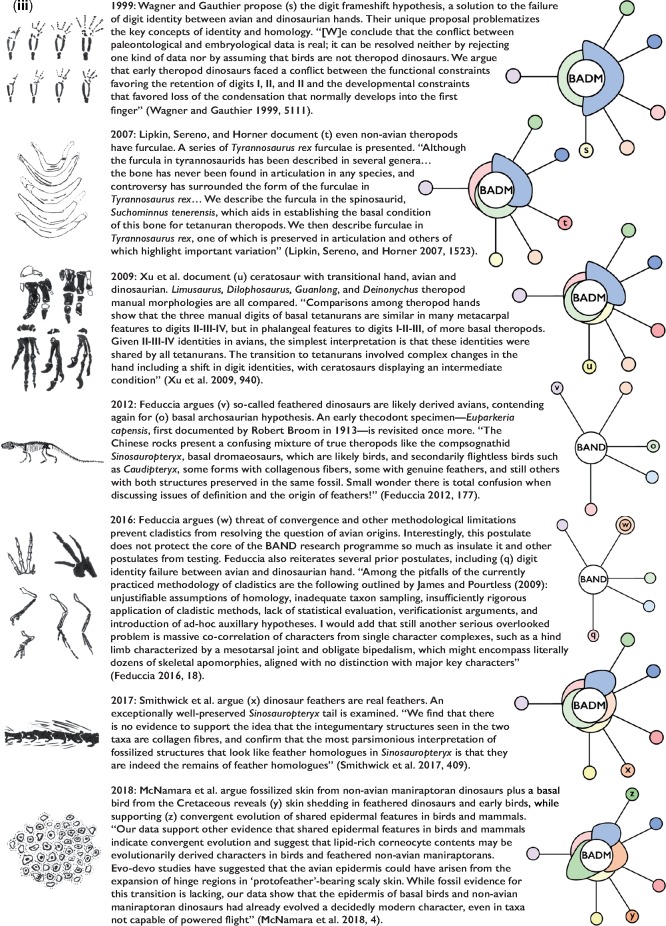
Graphics and illustrations by J.C.H. Most of the drawings are based on crucial specimens either depicted or discussed in the quoted text. The rest are conceptual representations of figures or notions from said text.

## Characterizing Progressive Practice

According to Lakatos, a scientific research program is a healthy one when it produces progressive problem-shifts (Lakatos 1968, 1970). This is his way of describing what happens as the core of a research program, along with its positive heuristic, suggests various hypotheses about testable circumstances which, when corroborated, add empirical content to the overall theory. This is how what Lakatos termed a protective belt grows around the core of a research program: empirical content is continually postulated and intermittently corroborated, leading to an overall increase in facts the theory has explained, predictions the theory has risked, and tests the theory has survived. A progressive research program is one that expands beyond its core, by continually postulating and at least intermittently corroborating additional empirical content. Finally, the status of a research program can change—what was once progressive can stagnate, even while the commitments of that program remain widely accepted.

**Figure 3.**
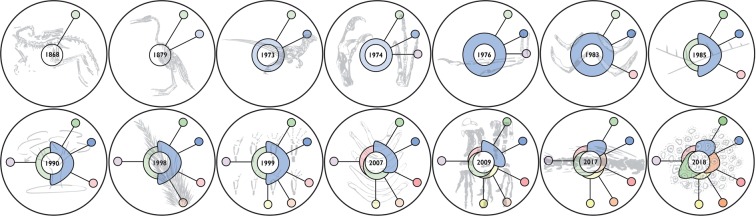
Early ideas proposed by Huxley (1868) and Williston (1879) found the BADM research program. Work by Ostrom (1973, 1974, 1976), however, is what begins to add a protective belt around the core commitment that birds are dinosaurs. Corroboration of ever more specific claims along particular lines of evidence causes the protective belt to swell in size. And as different lines of evidence are pursued and at least occasionally corroborated, protection from multiple, independent sources begin to build (indicated by different colors within the protective belt). Occasionally, speculative postulates are rejected; sometimes an opposing postulate is considered instead. Failures happen sometimes (postulates are proposed and then abandoned); still, especially since 1973, this looks like a progressive research program.

Since Ostrom began reviving the notion in 1969, the idea that “birds are dinosaurs” has emerged as the hard core of a progressive research program that has continually expanded the protective belt around its core—and expansion of that belt has occurred via the pursuit of multiple, distinct lines of postulation and corroboration leading to further, related postulations and corroboration. See Figure 3 for a chronological depiction of the Lakatosian model applied to BADM alone.

The BADM research program has added empirical content along multiple lines by, for instance: postulating that birds are not just dinosaurs but, more specifically, maniraptoran theropods (Smith et al. 2015); predicting that feathers evolved separately from flight (Clarke 2013); and surviving tests of that prediction via the discovery of feathered but flightless fossil dinosaurs (Xing et al. 2016). The BADM research program also includes additional, speculative empirical content that has not yet been fully corroborated—such as the digit frameshift hypothesis (Wagner and Gauthier 1999; Feduccia and Nowicki 2002). Finally, BADM has endured its unsuccessful postulates too: times when some of its speculative hypotheses have been falsified—such as those moments when proposed phylogenies have been revised or overturned (Gauthier 1986; James and Pourtless 2009; Baron et al. 2017), or when attempts to explain the evolution of flight from terrestrial ancestors have been challenged by the discovery of feathered, potentially volant, arboreal nonavian theropods (Padian 2001; Xu et al. 2003). It should be noted, however, that there is no consensus regarding the arboreal habits in microraptorine theropods. The jury is, so-to-speak, still out on whether these taxa may support a trees-down or ground-up scenario for the evolution of flight (Dececchi and Larson 2011). Regardless, this combination of empiricism, speculation, testability, falsification, and growth is precisely what constitutes healthy theoretical change during the robust progression of science. What the revived Lakatosian account uniquely and crucially allows us to do is to incorporate this sort of principled theoretical revision into a picture of rational theory choice in paleontological and other scientific practice.

## Characterizing Degenerative Practice

The Lakatosian account also allows us to rationally characterize unhealthy scientific practice. First, we should note that even a once-progressive research program can become degenerative, and vice versa; the status of a research program is not fixed. An unhealthy research program is just one that, according to Lakatos, does not produce or is no longer producing progressive problem-shifts: it is one that does not expand or is no longer expanding beyond its core. Since Lakatos’ account allows for some scientific speculation and failure, it can be difficult, and take quite some time, to identify when a research program is deteriorating. And because a progressive research program must both continually generate postulates and occasionally corroborate some of these postulates (adding empirical content in a protective belt around the hard core), there are at least two distinct ways in which a program can ail.

**Figure 4.**
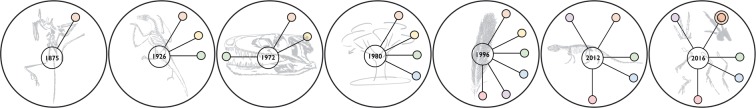
Owen (1875) founds the BAND research program but Heilmann (1926) brings it to prominence. However, even in the current era (during something of a heyday of fossil discovery), and despite the frequent proposal of speculative postulates by Feduccia (1980, 1996, 2012), no protective belt of empirical corroboration grows around the core commitment that birds are not dinosaurs, they are descended from some other group of more basal archosaurs instead. Prior postulates are repeated but neither corroborated nor extended. Interestingly, in recent work (Feduccia 2016), Owen’s initial postulate that birds and dinosaurs only look similar via convergence develops something of its own protective belt, constituted by the further claim that the threat of convergence prevents cladistics from ever settling the question of avian origins.

The first and more obvious mode of deterioration is the sort that occurs when a research program regularly generates speculative postulates, but corroboration of these postulates continually fails to occur, and so a protective belt never grows around the core. Lakatos calls this sort of research program degenerative rather than progressive. Insofar as the BAND research program has not lately grown beyond the claim—initially articulated in 1926 by Heilmann—that birds are descended from basal archosaurs, it looks as if BAND might be a degenerative research program of just this paradigmatically Lakatosian sort. See Figure 4 for a chronological depiction of the Lakatosian model applied to BAND alone.

The lack of a protective belt around the “birds are not dinosaurs” core does not mean that BAND is not a scientific research program: clearly, the program has an extended recent history of proposing speculative hypotheses (Feduccia 1980, 1996, 2002, 2012, 2013, 2016). It just means that corroboration of these hypotheses has repeatedly failed to occur, requiring perpetual retreat back to the basic claims of the core, around which no protective belt has grown, along with repetition of prior postulates.

It is worth noting here that a core commitment to the idea that birds are not dinosaurs was one that Heilmann himself was somewhat uncomfortable with, in that the simple lack of recognition of fossilized furculae in dinosaur remains known in the early 20th century essentially forced Heilmann, who adhered strictly to Dollo’s law of irreversibility, to argue against the otherwise overwhelming evidence that birds were in fact dinosaurs (Heilmann 1926). Nonavian dinosaur furculae have since been recognized in many species (e.g., *Tyrannosaurus rex*; Nesbitt et al. 2009), and though attempts have been made to increase the empirical content of the BAND research program by other means, these attempts have not succeeded (Prum 2002, 2003; Smith et al. 2015). Proposed speculations have necessarily become ever more radical, in order to support continued hypothesis generation and novelty. For instance, in the face of discoveries of feathered dinosaur fossils from China (Xu et al. 2003), which were originally interpreted as nonavian, BAND’s most vocal adherents have adopted a hypothesis in which these feathered dinosaurs are interpreted as birds with morphologies convergent with that of other dinosaurs, essentially abandoning the BAND hypothesis in favor of a radical rearrangement of our understanding of dinosaur phylogeny (Feduccia 2002). Proponents of this view strictly adhere to the axiom of Sir Gavin de Beer that “if it has avian flight feathers and avian flight wings, it’s a bird” (de Beer 1956). On this view, not only is the four-winged *Microraptor* a bird, but by default its feathered relatives, such as *Velociraptor mongoliensis*, the nonvolant predatory theropod of movie fame, must also be a flightless bird (i.e., Deinonychosauria, the clade that includes *Velociraptor,* would be nested within Avialae [birds], rather than the reverse). This is not a hypothesis that has garnered support among neontologists or paleontologists and it is worth noting that, previous to the discovery of vaned feathers in dromaeosaurs and other theropods (i.e., subsequent to the original description of feather filaments in these taxa), proponents of BAND argued vehemently that these taxa were totally unrelated to birds (Feduccia 2002).

## Characterizing Static Practice

But there is a second way in which a research program can turn out to be degenerative, in an expansive Lakatosian sense: it is not only that a program can fail to corroborate its speculative hypotheses; it can also cease to offer any such hypotheses at all. We propose this as an addendum to the Lakatosian model, and posit that it is better to call this sort of ailing research program static rather than degenerative. This is because it could be true, as some have suggested (Feduccia 2012), that birds descended from basal archosaurs, but also true that—given our precarious access to the contingent evolutionary history of the empirical world—no fossil evidence of this evolutionary trajectory either has been preserved or will ever be discovered. It would be a shame to declare a potentially true scientific commitment degenerative just because of our inability to access proof of that truth. Additionally, it might be true that cladistics cannot—in principle and with absolute certainty—ever entirely eliminate the threat of convergence to potentially mislead phylogenetic analyses, again, as some have suggested (Feduccia 2012, 2013, 2016). And it would be wrong to classify a potentially true claim about the limits of certain scientific methodologies as degenerative just because such a truth might be a vastly inconvenient one. Still, insofar as these sorts of claims—scientific as they may be—are deployed in order to explain the inability of a research program to either generate further speculative hypotheses or obtain corroboration, the best that such claims can do is justify an interminable lack of progress within the program to which they apply.

This sort of static research program might best be described as residing in an intermediate, purgatorial territory between progressive and degenerative programmes. Static science is not necessarily bad science: once-progressive research programmes can become static, for instance, when they become so established that they stop offering and corroborating new postulates. And in the absence of means for testing its postulates, a research program can be forced to go into stasis until technological or other developments allow for testing to begin or resume. Having a static research program might be better for a field of study than having a degenerative research program or having no research program at all. But such a program will not fare well in a field in which there is direct competition with a progressive research program. If this is indeed the current, comparative state of the BAND research program—if it is either straightforwardly degenerative, or has to resort to stasis (Feduccia 2002, 2013, 2016), in order to defend its lack of progress relative to that of the progressive BADM research program (Prum 2002, 2003; Smith et al. 2015)—then things do not bode well for the idea that birds are not dinosaurs. Even on a generous Lakatosian account that allows for principled theoretical revision, empirical content must be continually generated for ongoing research activity, and at least occasional corroboration is required for scientific progress.

## A Lakatosian Prediction

A key component of the currently entrenched dispute between BADM and BAND is the surprising frequency of appeals now being made, on both sides, to various philosophical figures and their ideas about what counts as scientific. David Hume and Arthur Schopenhauer as well as Popper and Kuhn have all made recent appearances in the debate, as have their ideas about skepticism, induction, falsification, paradigm shift, confirmation, and dismissal (Feduccia 2002, 2013, 2016; Prum 2002, 2003; Smith et al. 2015). Some philosophically minded scientists such as Charles Lyell and Carl Sagan have also been featured, along with their ideas about appearances and imagination (Feduccia 2016). But these metascientific moves have intensified rather than resolved the disagreement between BADM and BAND. So, here, we suggest a revival of our own—of Lakatos’ regrettably neglected account of rational theory choice and development—as a means of making progress in what has become a rather entrenched debate. Because such philosophical appeals can be similarly and unproductively made in other areas of highly contested science, we offer and revise Lakatos’ account in the hope that it might be helpful not just in this context but also elsewhere—wherever disputants in a scientific debate have begun to call into question whether their opponents even count as scientific, or are engaging in practices that are properly called scientific. The ongoing dispute about snake evolutionary history (Longrich et al. 2012; Caldwell et al. 2015) or, further afield, the debate about Clovis-era hunting and mammoth extinction (Grayson and Meltzer 2002) might each be candidates for application of the rehabilitated Lakatosian model.

Finally, one further and interesting result that follows from our application of the rehabbed Lakatosian model to the BADM versus BAND debate is the generation of the following pair of predictions: it is highly unlikely that either a straightforwardly degenerative or a static version of the BAND research program will flourish in paleornithology, without further generation of empirically confirmable speculative hypotheses, and at least occasional corroboration of those hypotheses. And given the competitive recent history of the BADM research program—with its continued generation of speculative hypotheses, and lately, its more than occasional corroboration—it is far more likely that this progressive research program, which considers birds to be maniraptoran theropod dinosaurs, will flourish instead. But this is a pair of speculative hypotheses designed to test the viability of the refurbished Lakatosian theory of progressive versus degenerative or static research programmes; we eagerly await either the refutation or the corroboration of these empirical postulates.
